# MMP-2 suppression abrogates irradiation-induced microtubule formation in endothelial cells by inhibiting αvβ3-mediated SDF-1/CXCR4 signaling

**DOI:** 10.3892/ijo.2013.1806

**Published:** 2013-02-04

**Authors:** DILIP RAJASEKHAR MADDIRELA, DIVYA KESANAKURTI, MEENA GUJRATI, JASTI S. RAO

**Affiliations:** 1 Departments of Cancer Biology and Pharmacology, University of Illinois College of Medicine at Peoria, Peoria, IL 61605, USA; 2 Pathology, University of Illinois College of Medicine at Peoria, Peoria, IL 61605, USA; 3 Neurosurgery, University of Illinois College of Medicine at Peoria, Peoria, IL 61605, USA

**Keywords:** angiogenesis, αvβ3 integrin, C-X-C chemokine receptor type-4, immunoprecipitation, matrix metalloprotease-2, radiation, stromal cell-derived factor-1, tumor conditioned media

## Abstract

The majority of glioblastoma multiforme (GBM) tumors recur after radiation (IR) treatment due to increased angiogenesis and IR-induced signaling events in endothelial cells (ECs) that are involved in tumor neovascularization; however, these signaling events have yet to be well characterized. In the present study, we observed that IR (8 Gy) significantly elevated MMP-2 expression and gelatinolytic activity in 4910 and 5310 human GBM xenograft cells. In addition, ECs treated with tumor-conditioned media (CM) obtained from IR-treated 4910 and 5310 cells showed increased microtubule formation. In view of this finding, we investigated the possible anti-angiogenic effects of MMP-2 downregulation using siRNA (pM.si) in IR-treated cells. We also determined the effect of CM obtained from mock, pSV (scrambled vector) and pMMP-2.si on endothelial cell growth and vessel formation. pM.si-CM-treated ECs showed inhibited IR-CM-induced SDF-1, CXCR4, phospho-PI3K and phospho-AKT and αvβ3 expression levels. *In vitro* angiogenesis assays also showed that the pM.si+IR decreased IR-induced vessel formation in ECs. Immunofluorescence and immunoprecipitation experiments indicated the abrogation of αvβ3-SDF-1 interaction in pM.si-CM-treated ECs when compared to mock or pSV treatments. External supplementation of either rhMMP-2 or rhSDF-1 counteracted and noticeably reversed pM.si-inhibited SDF-1, CXCR4, phospho-PI3K and phospho-AKT expression levels and angiogenesis, thereby confirming the role of MMP-2 in the regulation of αvβ3-mediated SDF-1/CXCR4 signaling. In addition to the *in vitro* results, the *in vivo* mouse dorsal air sac model also showed reduced angiogenesis after injection of pM.si alone or in combination with IR-treated xenograft cells. In contrast, injection of mock or pSV-treated cells resulted in robust formation of characteristic neovascularization. Collectively, our data demonstrate the role of MMP-2 in the regulation of SDF-1/CXCR4 signaling-mediated angiogenesis in ECs and show the anti-angiogenic efficacy of combining MMP-2 downregulation and IR when treating patients with GBM in the future.

## Introduction

Glioblastoma multiforme (GBM) is an aggressive, vascularized brain tumor that is characterized by high invasiveness. This tumor is capable of gaining resistance to various therapeutic agents as it utilizes alternative pathways once its primary signaling cascade is disrupted. Conventional chemotherapy, radiotherapy and immunotherapy are directed against tumor cells; in contrast, anti-angiogenic therapy is aimed at the vasculature of a tumor and will either cause total tumor regression or keep tumors in a state of dormancy (1). Angiogenesis is an invasive process involving the extracellular matrix and the proliferation and migration of endothelial cells (ECs). It is a prerequisite for tumor growth and metastasis formation (1). Therefore, understanding the cellular events involved in molecular regulation of the angiogenic events would provide novel therapeutic targets for the treatment of cancer.

For many solid tumors, radiation (IR) is the only treatment tool available that offers a clear survival benefit. However, even after exposure to IR, a cell population may manage to survive, either because it receives sub-lethal doses and/or because it successfully utilizes endogenous repair mechanisms (2). Molecular events in irradiated cells are altered, leading to upregulation of genes that favor cell survival and angiogenesis. Overexpression of proteases (e.g., MMPs) and cytokines and activation of surface receptors protect both tumor and non-tumor cells from apoptosis and increase their ability to trigger angiogenesis.

Anti-angiogenic agents may improve radiosensitivity by directly and/or indirectly inhibiting protective cell survival signaling pathways in both endothelial and tumor cells, resulting in increased apoptosis in both endothelial and tumor cells. Vascular targeting agents may act in an additive fashion to improve tumor response to radiation by decreasing overall tumor burden through vascular shutdown and by creating a tumor environment where maintaining viable, well-oxygenated tumor cells presents an ideal target for radiation therapy (3). The matrix metalloproteinases (MMPs) degrade the components of the extracellular matrix. Among the different factors involved in the acquisition of invasive capacity and angiogenic properties by tumor cells, the action of MMPs is critical. Earlier studies in our lab demonstrated direct evidence for a role of MMP-2 in tumor angiogenesis in which tumor cells express increased levels of MMP-2 and activate several key molecules leading to rapid cellular proliferation, increased motility, invasion and angiogenesis in lung cancer and gliomas (4–6).

Integrins are a family of cell-extracellular matrix adhesion molecules that play an important role in upregulating tumor associated blood vessels (7). Integrin αvβ3 has become an attractive target for drugs suppressing neovascularization from tumors and biomarker for tumor angiogenesis. Earlier studies have shown that blocking αvβ3 integrin induced EC death, implying its importance in pro-angiogenesis (8,9).

The membranous CXC chemokine receptor 4 (CXCR4) and its ligand, stromal-derived factor-1 (SDF-1), which is also known as CXCL12, are members of the chemokine family and their critical and active roles have been demonstrated in tumor growth and malignancy (10). In many tumors, metastasis is mainly related to SDF-1/CXCR4 (11). The most important sources of SDF-1 are bone marrow, lymph nodes, muscle and several regions of the central nervous system (12). Increased secretion or expression of SDF-1 in various organs in response to tissue damage such as IR, hypoxia or toxic agents has also been observed (13). Reports suggest that the SDF-1α/CXCR4 axis is involved in neovascularization and has also been detected in ECs (13). This implies that new therapeutic strategies aimed at blocking the SDF-1/CXCR4 axis could have important applications. Despite many scientific reports, the role of MMP-2 and irradiation-induced SDF-1/CXCR4 expression in regulating angiogenesis has not been completely elucidated.

IR continues to be the main, integral part of GBM treatment, but it is likely associated with intrinsic cellular resistance leading to rapid proliferation and high invasiveness. The role of MMPs in changing the cells to gain resistance to IR is a major therapeutic target. IR induces an increase in MMP-2 levels in almost all cancer types (5). IR-enhanced expression and activation of MMP-2 modifies tumor progression by altering the availability of various molecules that promote tumor angiogenesis. Enhanced MMP-2 secretion increases tumor survival by increasing angiogenic potential. These factors contribute to the high vascularity as well as radioresistance of GBM. Since SDF-1/CXCR4 is one of the major signaling axes of endothelial tubule formation and migration, especially in cancer, it is essential that we gain a better understanding of this phenomenon via αvβ3 and the signaling pathway that mediates radiation-inducible resistance. In this context, we attempted to study potential drug targets for radiosensitization using pMMP-2 siRNA in 4910 and 5310 human xenograft cell lines.

## Materials and methods

### Cell culture and reagents

Established human xenograft glioma cell lines 4910 and 5310 (kindly provided by Dr David James, University of California at San Francisco) are highly invasive in the mouse brain. These cell lines were generated and maintained in mice (14). These cells were cultured in RPMI-1640 medium (Mediatech Inc., Herndon, VA) supplemented with 10% FBS (Invitrogen Corp., Carlsbad, CA), 50 U/ml penicillin and 50 *μ*g/ml streptomycin (Life Technologies, Inc., Frederick, MD) in a CO_2_-chamber at 37°C. The human microvascular dermal endothelial cells (HMEC-1) were cultured in Advanced Dulbecco’s modified Eagle’s medium (Life Technologies) supplemented with glutamine, EGF and hydrocortisone (Stem Cell Technologies, British Columbia, Canada). Specific antibodies against MMP-2, SDF-1, CXCR4, PI3K, p-PI3K (Tyr 508), AKT, p-AKT (Ser 473), integrin αvβ3, GAPDH and HRP-conjugated secondary antibodies (Santa Cruz Biotechnology, Santa Cruz, CA) and Alexa Fluor-conjugated secondary antibodies (Life Technologies). We also used an αvβ3 integrin blocking antibody (Millipore Inc., Billerica, MA), recombinant human MMP-2 (rhMMP-2) (EMD Biosciences, San Diego, CA) and recombinant human SDF-1 (rhSDF-1) proteins (Pro Spec Bio, East Brunswick, NJ) in our study.

### Transient transfection and radiation

The specific MMP-2. siRNA (pM.si) and a scrambled sequence vectors (pSV) were designed and cloned as described earlier (15). At 70–80% confluence, 4910 and 5310 cells were serum-starved for 6 h after which they were treated with mock (1X PBS), pSV or pM.Si using X-tremeGENE HP DNA transfection reagent by following the manufacturer’s instructions (Roche Applied Science, Indianapolis, IN). The X-ray unit RS 2000 Biological Irradiator (Rad Source Technologies, Inc., Boca Raton, FL) operating at 150 kV/50 mA and delivering 0.71 Gy/min was used for radiation treatments. For the combination treatments, the culture medium was aspirated from mock-, pSV- or pM.si-treated culture plates and the cells were further treated with IR (8 Gy) or rhMMP-2 (25 ng/ml) or rhSDF-1 (25 ng/ml) and incubated for additional 12–16 h serum-free medium DMEMF-12 50/50 (Mediatech, Inc., Manassas, VA).

### Preparation of tumor conditioned media and gelatin zymography

Following transfection, the cells were cultured in RPMI-1640 serum medium for 24 h. At the end of experiment, the culture medium from mock-, pSV- or pM.si-treated culture plates was aspirated and serum-free DMEM-F-12 50/50 medium was added and incubated for another 12–16 h as above. The tumor conditioned media (CM) were collected from different treatments and quantified (6). Gelatin zymographic analyses were performed to determine the MMP-2 gelatinolytic activity using CM after different treatments as described previously (5). Further, angiogenic experiments were performed by culturing the endothelial cells (ECs) in CM obtained from various treatments as described above (4). After culturing in CM for 12–16 h, whole endothelial cell lysates were prepared and subjected to western blotting.

### Western blotting and immunoprecipitation

Western blotting and immunoprecipitation experiments were performed as described earlier (16). At the end of different treatments, the 4910, 5310 and ECs were gently washed with pre-chilled 1X PBS and whole cell lysates were prepared using RIPA buffer. Equal amounts of protein were fractionated on SDS-PAGE and immunoblotted with primary antibodies followed by incubation with species-specific, HRP-conjugated secondary antibodies. Signals were detected using ECL-enhanced Western blotting detection system (Amersham Pharmacia, Piscataway, NJ). For immunoprecipitation, equal amounts of protein (200 *μ*g) from ECs were immunoprecipitated with antibodies against SDF-1, αvβ3 and Nsp-IgG using μMACS protein G microbeads and MACS separation columns following the manufacturer’s protocol. The immunoprecipitates were subjected to Western blotting.

### In vitro angiogenic assay

Angiogenesis was performed as described earlier (17). Briefly, after transfection of 4910 and 5310 cells with mock, pSV or pM.Si, the cells were washed and incubated in serum-free medium for 16 h. For *in vitro* angiogenesis assay, the conditioned medium was collected and centrifuged to clear cellular debris. Approximately 4×10^4^ ECs were allowed to grow overnight in CM from 4910 and 5310 human xenograft cells in 96-well plates coated with Matrigel. After the incubation period, the formation of capillary-like structures was captured using a microscope attached to a CCD camera.

### Immunocytochemical and immunohistochemical analysis

Immunocytochemical and immunohistochemical analyses were performed as described previously (18). ECs were incubated in chamber slides for 16 h with the CM of 4910 and 5310 xenograft cells treated with mock or pSV or pM.Si with or without IR. The ECs were washed in PBS and fixed in 4% paraformaldehyde and permeabilized in 0.1% Triton X-100. Non-specific binding was blocked by BSA in PBS, followed by incubation with respective primary antibodies for 2 h at room temperature. The cells were washed and incubated with respective Alexa Fluor-conjugated secondary antibodies, subsequently mounted. Nuclei were counterstained with 4′,6-diamidino-2-phenylindole (DAPI). For immunohistochemical analysis, tissue sections (4–5 mm) (pSV or pM.Si with or without IR), were de-paraffinized in xylene, rehydrated in graded ethanol solutions, permeabilized in 0.1% Triton X-100 and incubated overnight at 4°C with anti-SDF-1 antibody. Slides were washed twice in PBS and incubated in HRP-conjugated secondary antibodies for 1 h at room temperature. The HRP-conjugated secondary antibody-incubated sections were washed and further incubated with DAB (3,39-diaminobenzidine) solution for 5–10 min while hematoxylin was used for nuclear counterstaining, mounted and photographed under a microscope.

### In vivo angiogenesis assay

*In vivo* angiogenesis assay was performed using the dorsal air sac model in athymic nude mice (nu/nu; 5–7-week old) as previously described (5). Initially, the mice were anesthetized by intraperitoneal injection of ketamine (50 mg/kg) and xylazine (10 mg/kg). Dorsal airsac was made by injecting 10 ml of air in the completely anesthetized mice. A 1.5–2.0-cm superficial incision was made horizontally along the edge of the dorsal air sac with the help of forceps and sterile diffusion chambers (Fisher, Hampton, NH) containing 4910 and 5310 cells (1.5×10^6^ cells) transfected with mock, pSV or pM.Si with or without IR were placed underneath the skin and carefully sutured. After 14 days, the animals were anesthetized with ketamine/xylazine and sacrificed by intracardial perfusion with saline (10 ml) and followed by 10 ml of 10% formalin/0.1 M phosphate solution. The tissue surrounding the implanted chambers was carefully resected and the chambers were removed from the subcutaneous air fascia. The air sac covering the chambers was photographed under visible light. The number of blood vessels within the chamber in the area of the air sac was counted and their lengths were measured. The Institutional Animal Care and Use Committee of the University of Illinois College of Medicine at Peoria (Peoria, IL) approved all surgical interventions and post-operative animal care. The animal protocol number is 858, May 27, 2009 and renewed on April 27, 2010.

### Statistical analysis

Data from at least three independent experiments were statistically analyzed using one way ANOVA and significant difference among various treatments were presented as mean ± SE at p<0.05 and p<0.01. Densitometric analyses was performed using ImageJ 1.42 (NIH, Bethesda, MD).

## Results

### pM.Si-CM of human xenograft cell lines downregulated IR-induced angiogenesis

**Figure 1:**
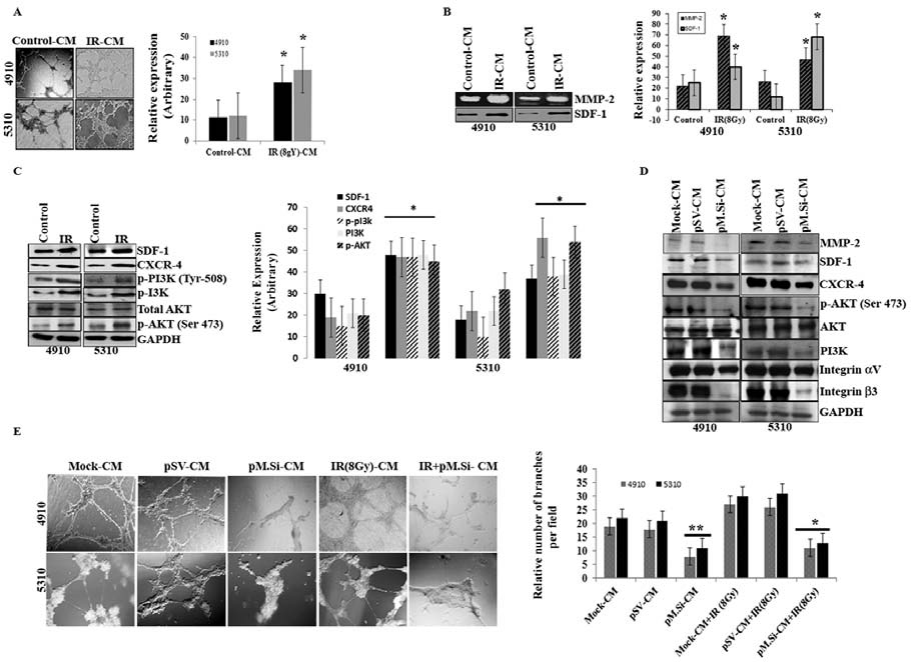
pM.Si-CM downregulates IR-induced angiogenesis in human xenograft cell lines 4910 and 5310. Cells (4910 and 5310) were treated with mock, pSV or pM.Si alone or in combination with IR (8 Gy) as described in Materials and methods. (A) *In vitro* angiogenesis. The conditioned media was added to 96-well plates that were coated with Matrigel and pre-seeded with human dermal microvascular endothelial cells (ECs) (2×10^4^ cells/well). After overnight incubation at 37°C, cells were observed under the bright field microscope for the formation of capillary-like structures. The degree of angiogenic induction by mock- and IR-CM were quantified for the numerical value of the product of the relative capillary length and number of branch points per field and indicated in a bar diagram. The data are presented as the mean ± SE of three independent replicates with significance denoted by ^*^p<0.01. (B) Gelatin zymography (MMP-2) and western blot analysis (SDF-1) were performed. The experiments were carried out thrice and the data are presented as the mean ± SE of three independent replicates with significance denoted by ^*^p<0.01. (C) Seventy-two hours post-transfection, xenograft cells were harvested and whole cell lysates were prepared using RIPA buffer. Whole cell lysates were subjected to western blotting for SDF-1, CXCR4, p-PI3K (Tyr 508), PI3K, AKT and p-AKT (Ser 473). GAPDH was used to confirm equal loading. The data are presented as the mean ± SE of three independent replicates with significance denoted by ^*^p<0.01. (D) ECs were grown on the mock-, pSV- and pM.Si-CM for 16 h. Cells were then collected and whole cell lysates were subjected to western blotting for MMP-2, SDF-1, CXCR4, p-AKT (Ser 473), AKT, PI3K and integrin αvβ3. The blots were stripped and re-probed with GAPDH antibody as an internal control for the respective proteins. (E) *In vitro* angiogenesis was performed under similar conditions as described for (A). The degree of angiogenic induction was quantified for the numerical value of the product of the relative capillary length and number of branch points per field. The data are presented as the mean ± SE of three independent replicates with significance denoted by ^*^p<0.05 and ^**^p<0.01.

Our previous study (5) suggests that the infiltrative behavior and the survival mechanisms of glioma have a definite relationship to MMP-2 expression and IR exposure. Therefore, we investigated the ability of pM.Si to prevent this characteristic aggressive angiogenesis following radiation. The results of *in vitro* angiogenesis assay (Fig. 1A) indicated that there was a marked rise in the number of capillary-like endothelial tube structures when treated with IR (8 Gy)- of 4910 and 5310 cells as compared to control-CM.

Because MMP-2 activity was thought to be necessary for endothelial tubule formation, we next examined whether IR altered MMP-2 activity. This effect was measured by gelatin zymography and determined to function in a dose-dependent manner (data not shown). At a dose of 8 Gy, MMP-2 activity was upregulated in IR-treated 4910 and 5310 cells compared to control-CM (Fig. 1B). We next examined the relative expression of SDF-1 with IR in the CM of both cell lines. SDF-1 was significantly upregulated in IR treatment as compared to control in 4910 and 5310 cells (Fig. 1B). When whole cell lysates were subjected to western blotting, there was remarkable increase in the expression levels of SDF-1, CXCR-4, p-PI3K (Tyr 508), PI3K, AKT, p-AKT (Ser 473) in IR treated cells, compared to controls (Fig. 1C).

As SDF-1 is known to regulate the morphogenesis of ECs (19), we next proceeded to verify the effect of pM.Si-CM in downregulating SDF-1 expression as well as CXCR4 and downstream signaling molecules in ECs. We previously demonstrated that transcriptional knockdown of MMP-2 in tumor cells inhibits secretion of SDF-1 (20). pM.Si-CM abrogated the expression of SDF-1 and CXCR4 when incubated with ECs for 16 h (Fig. 1D) and this reduction correlated with reduced expression of MMP-2 in ECs. Because several studies indicate that PI3K/AKT mediates the activation of angiogenesis, we determined the effect of MMP-2 suppression on PI3K/AKT. As shown in Fig. 1D, pM.Si-CM inhibited the expression of PI3K and phosphorylation of AKT (Ser 473) as compared to mock- and pSV-CM in ECs.

Studies indicate direct interaction between MMP-2 and integrin αvβ3 and their coordinated interplay is essential to endothelial tubule formation (17,21,22). Based on this information, we investigated the role of pM.Si-CM on MMP-2 and integrin αvβ3 on ECs in MMP-2 regulated SDF-1 expression in 4910 and 5310 cells. Western blot analysis revealed (Fig. 1D) αvβ3 suppression by pM.Si-CM, thereby indicating the critical role of MMP-2 in angiogenesis. Since IR is a vasculogenic stimulus, we investigated the effect of radiation on endothelial cell capillary-like network formation. MMP-2 promotes tumor vascularization and, in turn, renders the tumor cells resistant to radiotherapy. pM.Si-CM with or without IR from 4910 and 5310 cells failed to induce capillary network formation when added to ECs. On the other hand, mock- and pSV-CM triggered the angiogenic process, which is evident from the increase in the percentage of branching when compared to non-irradiated counterparts (Fig. 1E).

### pM.Si-CM inhibited IR-induced PI3K/AKT expression and angiogenesis in ECs and supplementation of rhMMP-2/rhSDF-1 reverses inhibition

**Figure 2:**
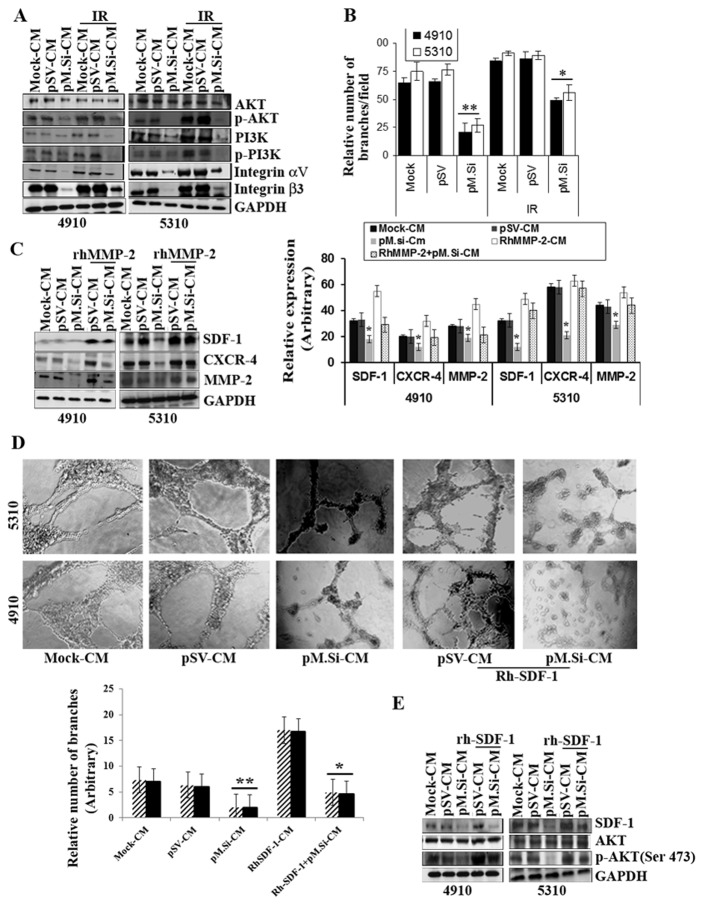
pM.Si-CM inhibits IR-induced PI3K/AKT expression and angiogenesis in ECs and supplementation of rhMMP-/rhSDF-1 reverses inhibition. (A) ECs were grown in mock-, pSV- and pM.Si-CM with or without IR. The whole cell lysates were subjected to western blotting to check the expression levels of AKT, p-AKT, PI3K, p-PI3K, integrin αv and integrin β3 using specific antibodies. The blot was restriped and GAPDH was used as a loading control. (B) *In vitro* angiogenesis was done with overnight incubation of ECs at 37°C with mock-, pSV-, IR (8 Gy)-CM or pM.Si alone or in combination with IR (8 Gy). The cells were observed under a bright field microscope for the formation of capillary-like structures. The degree of angiogenic induction was quantified for the relative capillary length and number of branch points per field. The data are presented as the mean ± SE of three independent replicates with significance denoted by ^*^p<0.05 and ^**^p<0.01. (C) ECs from 4910 and 5310 cells were grown in the presence of mock-, pSV- or pM.Si-CM with or without IR (8 Gy) for 16 h. pSV- and pM.Si-CM were treated with 25 ng/ml rhMMP-2. Whole cell lysates of ECs were prepared at the end of 16-h treatment and subjected to western blotting to check the expression levels of SDF-1, CXCR4 and MMP-2 using specific antibodies. The blot was restriped and GAPDH was used as a loading control. The data are presented as the mean ± SE of three independent replicates with significance denoted by ^*^p<0.05 and ^**^p<0.01. (D) *In vitro* angiogenesis was carried out under similar conditions as noted in Fig. 1A and is representative of at least three independent repetitions. RhSDF-1 (25 ng/ml) was added to pSV- and pM.Si-CM and incubated for 16 h. The degree of capillary network formation is indicated in a graph. The data are presented as the mean ± SE of three independent replicates with significance denoted by ^*^p<0.05 and ^**^p<0.01. (E) Endothelial cells were treated with pSV-CM or pM.Si-CM supplemented with rhSDF-1 (25 ng/ml) for 16 h. The whole cell lysates were subjected to western blot analysis for the expression of SDF-1, AKT and p-AKT with their respective antibodies and GAPDH was used as the loading control.

Our results show that pM.Si-CM with or without IR diminished the expression of protein tyrosine kinase PI3K and phosphorylation of PI3K and phosphorylation of AKT (Ser 473) in ECs compared to mock- and pSV-CM (Fig. 2A). A recent study suggested that IR upregulates αvβ3 expression in ECs and consecutively phosphorylates AKT (Ser 473), which may provide a tumor escape mechanism from radiation injury mediated by integrin survival signaling (23). In view of this, we investigated the role of pM.Si-CM on αvβ3 expression in ECs. We demonstrated that αvβ3 was downregulated by IR-induced pM.Si-CM as compared to mock-CM- or pSV-CM-treated ECs (Fig. 2A). Further, our results show that pM.Si-CM combined with IR potentially reduced endothelial network formation, which indicates a strong relationship between IR-induced MMP-2 and αvβ3 downregulation with respect to angiogenesis in ECs. Fig. 2B shows an angiogenic graph indicating the efficacy of pM.Si-CM obtained from 4910 and 5310 cells, in downregulating the number of capillary network branches per field and reveals reductions ≤80 and 73%, respectively, compared to mock- and pSV-CM. In contrast, pM.Si-CM combined with IR demonstrated ≤40 and 45% reduction in the number of capillary formation in 4910 and 5310 cells, respectively, which shows the efficacy of pM.Si-CM in combination therapy. To further assess the role of rhMMP-2 in SDF-1 expression, rhMMP-2 recombinant protein was added to the CM of pM.Si and pSV and incubated for 12–16 h in ECs. There was remarkable elevation of SDF-1 and CXCR4 expression in ECs observed in pM.Si-CM-treated cells as shown in Fig. 2C. These findings suggest that MMP-2 upregulates the expression of SDF-1 and CXCR4 in ECs as also shown in the graph (Fig. 2C). To assess further, the pM.si-CM inhibited SDF-1 expression, we elevated the SDF-1 by rhSDF-1 in pSV-and pM.Si-CM of both the cell lines in ECs. The addition of rhSDF-1 to pM.Si-CM-treated ECs reversed pM.Si-CM-mediated inhibition of capillary network formation by ECs (Fig. 2D). These results suggest that MMP-2 downregulation inhibits SDF-1 expression and secretion, which results in impaired endothelial tubule formation. Additionally, western blot analysis established that the supplementation of rhSDF-1 restored SDF-1 mediated elevation of AKT phosphorylation, which was inhibited by pM.Si-CM from both 4910 and 5310 cells and indicates AKT-mediated angiogenesis (Fig. 2E). Thus, rhSDF-1 elicited a rapid and robust increase in the expression of pM.Si-CM-abrogated pro-angiogenic molecules and endothelial capillary network formation.

### Knockdown of MMP-2 by pM.Si-CM inhibits IR-induced SDF-1 expression via integrin αvβ3 in ECs

**Figure 3:**
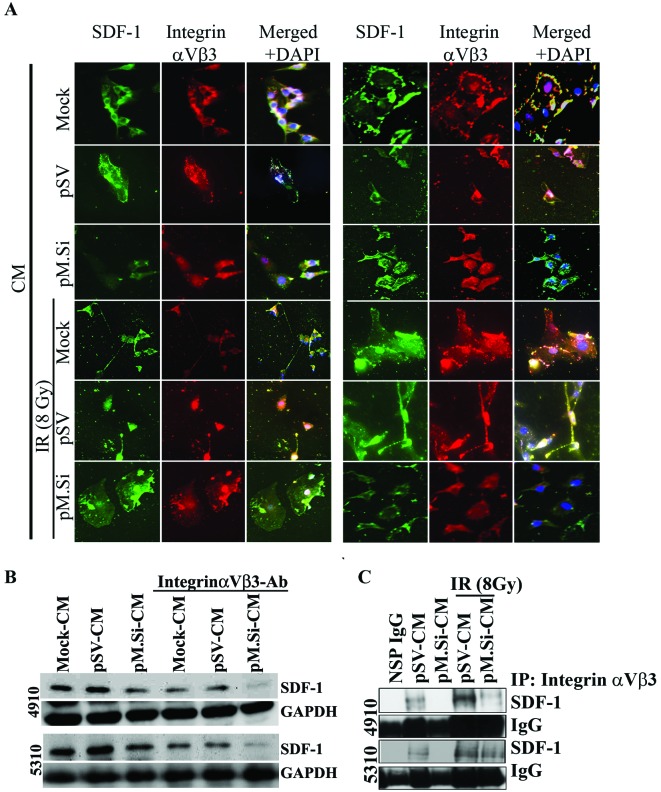
Knockdown of MMP-2 by pM.Si-CM in ECs inhibits IR-induced SDF-1 expression via integrin αvβ3. Knockdown of MMP-2 inhibits integrin αvβ3-mediated SDF-1 expression in ECs. (A) ECs were plated in 4-well chamber slides (4×10^3^ cells/well) and grown in mock-, pSV- or pM.Si-CM of both 4910 and 5310 cells either alone or combined with IR (8 Gy) for 16 h. Cells were fixed, permeabilized and incubated with antibodies specific for integrin αvβ3 and SDF-1 (1:100 dilution) for 2 h at room temperature followed by Alexa Fluor secondary antibodies for 1 h, stained with DAPI and mounted. Randomly selected microscopic fields of three independent experimental replicates are shown. (B) EC lysates were collected and lysed after 16 h of incubation in mock-, pSV- or integrin αvβ3 blocking Ab mock- or integrin αvβ3 blocking Ab pM.Si-CM from 4910 and 5310 cells. Western blot analysis performed for SDF-1 using a specific antibody. GAPDH was used as a loading control. (C) ECs were grown on pSV-, pM.-Si-CM, with or without IR (8 Gy) for 16 h. The ECs of whole cell lystates (200 *μ*g) were immunoprecipitated with antibodies against NSP-IgG and integrin αvβ3 using μMACS protein G microbeads and MACS separation columns. Immunoprecipitate was subjected to western blotting using SDF-1 specific antibody.

Understanding how endothelial MMP activity is involved in the angiogenic phenotype has enormous implications on cancer therapy since angiogenesis is necessary for tumor growth and metastasis. Immunofluorescence analysis of ECs revealed that co-localization of SDF-1 and integrin αvβ3 decreased in pM.Si- or IR-induced pM.Si-CM compared to mock- and pSV-CM from 4910 and 5310 cells (Fig. 3A). The essential functional role of SDF-1/αvβ3 interaction with pM.Si-CM of 4910 and 5310 cells was further verified by treating CM with a function-blocking anti-αvβ3 integrin antibody. The supplementation substantially inhibited SDF-1 expression in ECs treated with mock- and pSV-CM. pM.Si-CM along with blocking anti-αvβ3 integrin antibody further decreased SDF-1 expression compared to mock- and pSV-CM anti-αvβ3 integrin-treated controls as shown in Fig. 3B. We next examined their expression levels and their interaction using co-immunoprecipitation. We observed a significant decrease in the expression levels of SDF-1 in ECs treated with pM.Si-CM of both cell lines. The αvβ3 binds to SDF-1 in ECs treated with mock- and pSV-CM from 4910 and 5310 cells and facilitates the activation of pI3K/AKT signaling. On the other hand, ECs treated with pM.Si showed decreased binding of αvβ3 to SDF-1 (Fig. 3C), implying the critical role of MMP-2 in recruiting SDF-1 to αvβ3 and promoting angiogenesis.

### Immunocytochemical and immunohistochemical analyses

**Figure 4:**
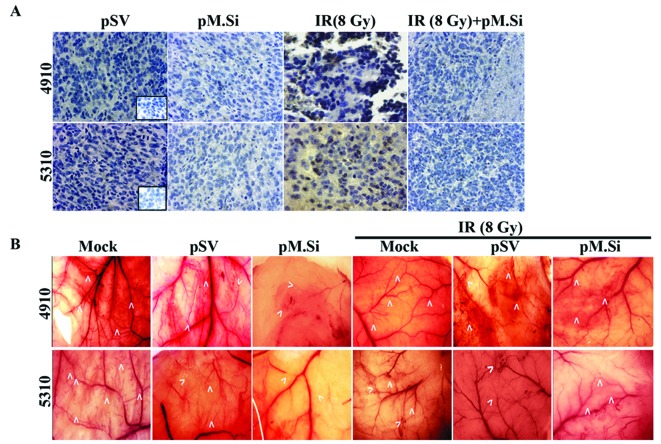
MMP-2 knockdown abrogated SDF-1 expression *in vivo*. (A) Immunohistochemistry was performed for expression of SDF-1 using specific antibody. Data shown are representative fields (×40). Also shown is the negative control where the primary antibody was replaced by non-immune serum (inserts). (B) *In vivo* angiogenic assay was completed using the dorsal air sac model as described in Materials and methods. Briefly, the animals were implanted with diffusion chambers in a dorsal cavity containing mock, pSV or pM.Si-transfected 4910 and 5310 cells treated with or without IR (8 Gy). Ten days after implantation, the animals were sacrificed and the number of new blood vessels covering the diffusion chamber was observed under a bright field microscope for the presence of tumor-induced neovasculature and pre-existing vasculature. Implantation of a chamber containing mock, pSV or IR (8 Gy) in 4910 and 5310 cells resulted in the development of microvessels (arrows) with curved thin structures and many tiny bleeding spots. In contrast, implantation of pM.Si with or without IR (8 Gy) resulted in a decreased number of microvessels.

We next sought to determine the effect of pM.si alone or in combination with IR on SDF-1 and angiogenesis *in vivo*. The brain tumor sections were prepared as described earlier (5). pSV and pM.Si alone or in combination with IR were evaluated histologically for expression levels of SDF-1 (Fig. 4A). In pSV treated tumor section a high expression of SDF-1 was noted compared to pM.Si alone or combined with IR treatment. These *in vivo* results corroborate with our *in vitro* results.

### pM.Si treatment suppresses angiogenesis in vivo

To confirm our *in vitro* findings, we examined whether suppression of MMP-2 could inhibit tumor angiogenesis using the dorsal air sac assay. Implantation of a chamber containing 4910 and 5310 cells as described in Materials and methods resulted in the formation and development of new blood vessels (Fig. 4B) with curled, thin structures and many tiny bleeding spots in addition to the pre-existing vessels. However, a marked reduction in the development of such tumor-induced microvasculature was observed in the pM.Si treatments (with or without IR) in 4910 and 5310 cells as compared to mock and pSV treatments.

## Discussion

After radiation treatment of GBM primary tumors, patients are at high risk of metastatic invasion due to extensive angiogenic process (24). These patients could benefit from the addition of MMP-2 inhibitors to standard radiotherapy. Our study was designed to examine the effects of combining MMP-2 inhibition and radiation on human xenograft cells and their conditioned media (CM) on microtube formation of endothelial cells (ECs) *in vitro* and *in vivo*. Our results demonstrate that MMP-2-depleted-CM abrogated IR-induced SDF-1/CXCR4 expression and PI3/AKT-mediated angiogenesis in glioma xenograft cells. We also showed MMP-2 downregulation led to weakened interaction of MMP-2 with integrin αvβ3, thereby enhancing endothelial inhibition of microtubule formation.

Altered proteinase activity was observed in several studies following irradiation of tumor cells and tissues. Upregulation of MMP-2 subsequent to different irradiation conditions was observed in different tumor types (e.g., pancreatic cancer, glioblastoma, colorectal cancer and fibrosarcoma), leading to enhanced cell invasion (25–31). Gelatinases have been identified as major factors for high-grade gliomas and their expression is directly correlated to the type of malignancies (28). However, the cellular and molecular events that promote GBM are not completely understood. Since GBM cells are capable of switching their dependency from one signaling pathway to an alternative pathway, it is necessary to impede the gelatinase activity of MMP-2, which is the main cause of diverse mechanisms for GBM survival. Several studies have indicated the angiogenic process is mostly related to alterations in the gelatinolytic activity of MMPs (28). Even though tumors invariably recur within the radiation field, radiotherapy is a mainstay in the treatment of GBM. Any method that improves local control of the tumor by radiotherapy would improve the curability of patients with GBM (32). Here, we propose that the cure rates for this malignancy might be improved by targeting MMP-2-mediated SDF-1/CXCR4 expression and pathway, thereby preventing reconstitution of tumor vasculature following radiation.

As an initial step of our study, we observed that tumor-conditioned medium from irradiated 4910 and 5310 cells enhanced capillary tube formation in ECs as compared to control-CM, which correlated with high MMP-2 and SDF-1 expression levels. MMP-2 upregulation led to increase in CXCR4, PI3K and phosphorylation of AKT (Ser 473) as compared to mock and pSV in 4910 and 5310 cells. We investigated whether MMP-2-downregulated CM by pM.Si. plays any role in curbing the molecular and morphological events in ECs. pM.Si-CM alone or in combination with IR was capable of inhibiting the upregulation of angiogenic molecules to a significant extent. This result correlated with the results of our *in vitro* angiogenesis assay; Matrigel assay revealed that CM of pM.Si with or without IR showed impaired growth and enhanced branching distortion of ECs compared to a highly branched network observed in mock-and pSV-CM with or without IR. Upregulation of MMP-2 by IR paves the way for degrading the underlying basement membrane and provides a route for sprouting ECs. Similarly, IR-CM has generated extensive tube formation on ECs, which was accompanied by increased secretion/expression of SDF-1. The new microvessels formed in tumor angiogenesis are abnormal and remain leaky and demonstrate other morphologic abnormalities as they lack a properly formed basement membrane (33,34). In contrast, IR in combination with pM.Si effectively suppressed endothelial tube formation as compared to mock- and pSV- CM. This substantiates the role of pM.Si-CM on MMP-2-generated angiogenesis.

CXCR4 is recognized as the most commonly expressed receptor on many types of cancer cells. Its upregulation has been associated with metastasis and its suppression is accompanied by inhibition of SDF-1-induced invasion of tumor cells (35). It has been demonstrated that low doses of IR increase the expression of chemokines along with CXCR4 in human ECs (36). SDF-1/CXCR4 signaling is one of the major GBM survival factors because it enhances endothelial tubule formation. Our results indicate that CM of both cell lines treated with pM.Si abrogate IR-induced SDF-1/CXCR4 expression on ECs compared to mock-CM and pSV-CM.

In our study, knockdown of MMP-2 at the transcriptional level also prevented IR-induced phosphorylation of AKT and PI3K as supported by distorted microtububle formation *in vitro*. Moreover, supplementation of rhMMP-2 or rhSDF-1 reversed the effect of pM.Si. The downstream molecules PI3K and AKT reversed their expression levels after rhMMP-2 or rhSDF-1 supplementation in pM.Si-treated CM, thereby confirming that molecular modulations are due to the critical role of the pM.Si-mediated effect.

In the present study, we have established that SDF-1/CXCR4 induced downstream signaling of PI3K and AKT. This event might have generated more secretion of SDF-1, which in turn, activated more MMP-2 exogenously. The PI3K/AKT pathway is involved in prototypical endothelial functions including the regulation of angiogenesis. In ECs, phosphoinositide 3-kinases are activated downstream of several receptors, including G-protein-coupled receptors (e.g., chemokine receptors), tyrosine kinases (e.g., vascular endothelial growth factor receptors), integrins and death receptors (e.g., TNFα receptor). In turn, phosphoinositide 3-kinase signaling promotes nitric oxide release (through endothelial nitric oxide synthase phosphorylation), angiogenesis (through RhoA), endothelial progenitor cell (EPC) recruitment and cell viability and increasing evidence suggests that these angiogenic stimuli are modulated by ionizing radiation (37–40).

Blood vessel growth is regulated by angiogenic CXC chemokines and radiation is a vasculogenic stimulus. IR induces DNA damage in the nucleus, triggering a large network of intracellular signaling events. These include transient activation of pro-survival pathways and PI3K/AKT signaling as well as upregulation of the expression of the chemokine receptor CXCR4. Low doses of radiation increase the expression of chemokines, including CXCR4, in human ECs (36). We investigated the effect of IR on endothelial cell chemokine signaling, receptor expression and endothelial network formation. The downstream signaling networks play an important role in tumor radiosensitivity by eliciting pro-survival and pro-inflammatory responses. The PI3K/AKT pathway, which is constitutively activated in various cancers, plays a critical role in promoting endothelial cell growth.

Integrins are extracellular matrix receptors involved in angiogenesis. In order for angiogenesis to occur in IR-treated or untreated xenograft cells, the binding of SDF-1 to its receptor CXCR4 must occur. This leads to activation of ECs and results in the upregulation of specific integrin receptors on the cell surface, such as integrin αvβ3, as compared to mock and pSV xenograft cells. Many studies have indicated that expression of integrin αvβ3 is upregulated on the surface of proliferating ECs in angiogenic microvessels, including those in glioblastoma (grade IV malignant astrocytoma) (41–43). We found that IR induced expression of αvβ3 in ECs. We analyzed the combinational *in vitro* effects of IR (8 Gy) and pM.Si-CM by measuring endothelial αvβ3 integrin expression. First, immunocytochemical analysis revealed diminished integrin signaling with SDF-1 when treated with pM.Si-CM with or without IR in ECs in comparison with mock- and pSV-CM. IR upregulated αvβ3 expression and activated AKT in ECs, thus forming a defense mechanism and survival signal against IR damage (23). However, this effect was counteracted by pM.Si-CM when combined with IR as compared to mock- or pSV-CM. Second, treatment with pM.Si-CM resulted in the inhibition of IR-induced AKT phosphorylation and enhancement of IR-induced endothelial tubule formation *in vitro*. Third, pM.Si with or without IR-CM treatment further impaired vascular morphogenesis significantly. Finally, our *in vivo* angiogenesis assay results substantiated the role of pM.Si in preventing neovascularization.

The expression of MMP-2-mediated SDF-1/CXCR4 in ECs was markedly attenuated in the presence of pM.Si-CM alone or pM.Si-CM with IR (8 Gy). Based on the results of the present study, we conclude it is conceivable that αvβ3 integrin-mediated angiogenesis mechanisms after radiation toxicity can effectively be interrupted by co-administration of pMMP-2 siRNA.
